# Our New York Letter

**Published:** 1889-10

**Authors:** M. B. Hutchins

**Affiliations:** New York


### OUR NEW YORK LETTER.

NEW YORK, September, 1889.

Rhinoplasties are not uncommon, and consequently it is not often that they are reported in detail. I hope your readers will therefore pardon the liberty taken in describing the operation as recently seen. The patient, a woman aged 68, had an epithelioma, the center of which was in the right ala of the nose, the growth extending peripherally about half an inch (including infiltrated and cicatricial tissue). Instruments, having been previously cleaned and disinfected, were placed in carbolic solution 1–100. Cat-gut and silk-worm gut had been rolled on glass spools and placed in little jars containing alcohol, having a perforated rubber stopper for use during operation. "Thiersch's solution" (Ac. Salicyl. ʒi, ac. Boric ʒvi, Aquae Oij) was prepared for irrigation. Operator's and assistant's hands were thoroughly washed and disinfected, and again washed so often as required during the operation. Nose and forehead of patient were thoroughly washed with bichloride sol. 1–2500 and then with "iodoform ether" five per cent. Towels wet with the bichloride solution were used to bind the hair back and to surround adjacent portions which the hands of the operators might touch. The posterior nares were tamponed to prevent the flow of blood into the larynx.

An incision was made to include the whole of the infiltrated tissues together with the cicatrices left by the disease (the cicatricial tissue of cancer being known to contain a limited member of cancer cells). Underlying cartilaginous portion of nose smoothly cut away with C. scissors. The whole of the right alar portion of nose was removed, skin partly dissected from osseous portion and cut away, and about one-third of left alar portion removed.

A pattern of the flap desired was made by adapting a piece of oil silk and cutting a fit with slight allowance for shrinkage of flap. Flap was then taken from region between and above superciliæ (which had previously been shaven, at inner third and washed with disinfectants) inclining to right frontal eminence. Flap was about 1½ inches wide above and half an inch wide below, forming pedicle. Pedicle formed just anterior and internal to inner canthus of eye, including the angular artery. Flap was thus brought around and down (loosely turned on pedicle) to cover exposed nasal openings. The fit was good and there was only a semi-blanching of the flap, while bleeding was sufficiently free from lower border. Edges now stitched with silk-worm-gut and cat-gut to adjacent cut surfaces, and the first stage of nose-formation was complete, though said nose could scarcely be said to belong to the things beautiful. The wound left in forming the flap received two stitches at upper part, remainder left to fill with blood for "organization." Irrigation with Thiersch's sol. was frequently employed during the operation. Sponges which fell upon the floor were not permitted to be again used during the operation. The wound on forehead was dressed with iodoform gauze and a bandage. Sutured edges of nose were covered with iodoform powder, fixed edge of flap with a strip of gauze along the border, and then a loose "curtain" of gauze let fall over nose—so there would be no added interference, through bandage, with the feeble circulation. Nostril edge of new nose was an eighth of an inch longer than corresponding old border of left side, being left to retract and turn in for formation of the natural curved or rounded edge.

One rather novel feature of the operation was the use of chloroform, and the patient, after being anæsthetized, being placed in a sitting posture, in which position the operation was performed. Only enough chloroform was then given to keep patient quiet. patient showed no tendency to collapse. Bleeding continued, both from flap and forehead wound, for about six hours. This was as desired of the flap, to prevent stasis. Temperature has not exceeded 101 F., and though flap was somewhat purplish, and slightly offensive in new nostril, the circulation seems sufficient and warmth enough is present to justify the belief that the operation will prove successful. Should the end of flap slough, the defect will be supplied at the expense of adjacent cheek. Sutured edges are kept covered with iodoform powder, and the bandage at root of nose wet with T—'s solution.

A patient, who had had a rhinoplasty performed for epithelioma, came to the Skin and Cancer Hospital with a recurrence involving both the "new" nose and adjacent tissues, forming an opening as large as the thumb. Recently the infiltrated glands of submaxillary region were removed, with the hope of checking extension of the disease. At the time the "new nose" was made, the forehead wound was covered with skin transplanted from the arm, and the "grafting" was successful.

Apropos of the use of white precipitate ointment in psoriasis, it may be of interest to mention the rather unexpected, though known, effect in a case thus treated for a general outbreak of the disease. The application ("1—4") acted beautifully on the skin, but one morning the patient waked up with symptoms of quite severe mercurialism—throat, tonsils and gums being affected; teeth became loose, and characteristic mercurial fœtor was present in the breath. Under usual treatment for mercurialism, symptoms (save pain in lower jaw and looseness of the teeth) subsided. At first the applications were entirely suspended, but after two days the ointment was again applied, and the symptoms were aggravated. The visiting physician considers patient's skin possessed of unusual absorptive powers. The skin lesions continue to fade under equal parts ungt. picis et. zinc. ox. and ungt. o. z.

Two laparotomies, seen within the past month, which were performed under antiseptic precautions, showed rather unexpected action in the healing of abdominal wound. The first suppurated at site of the drainage tube (glass), which had been left in position for the first two days. The second healed by "first intention," despite the fact that an assistant who came in during the operation, put his hands on the wound without having previously washed them.

One of those chronic "varicose ulcers" of the leg which are so troublesome to heal improved fifty per cent. in a week under usual bandaging, washing with two and a half per cent. carbolic acid and a dressing of iodoform gauze. In fact, it seems that any disease the dermatologist meets is best treated by something possessing antiseptic properties.

The confusion of dermatology is not the less confounded by a number of cases recently appearing at the Skin and Cancer Hospital and variously diagnosed lichen ruber, lichen ruber planus, lichen planus and the lichen ruber pilaris of Devergie. There is no doubt that a diverting local fight will occur over this class of cases, made a little more lively by an occasional gun from Europe. A student of skin diseases will find any amount of confusion if he reads a number of text-books and a few journals. It would seem that there is only one way to keep a clear nomenclature, and that is to "swear by" one another! But this would be narrow, and so the microscope will probably prove the guide to lead us out of the wilderness.

The most extensive case of trichophytosis (ringworm) corporis seen at the Skin and Cancer Hospital was in a middle-aged German who had had the disease for two years. Beginning originally in scroto-femoral region, it involved the anterior, internal and posterior aspects of both thighs, extending thence over abdomen and buttocks. Entire neck, to upper borders of clavicles and scapulæ involved, and a few patches on forearms, lower legs and backs of feet. He made the surprising statement that neither his wife nor children had contracted the disease.

Another interesting case, also in a German, was an attack of tinea versicolor, implicating skin of neck, arms and body; present in varying degrees for a number of years. He was treated with baths and a lotion containing bichloride of mercury, about gr. one and one quarter to the ounce.

M. B. HUTCHINS, M. D.

